# Gold Caps

**Published:** 1874-02

**Authors:** 


### GOLD CAPS.

Gold caps, to be used in connection with oxy-chloride of zinc, Hill's Stopping or any similar plastic filling, have been devised, made und used by Dr. J. E. Fisk, of Salem, Mass.

The caps are made complete by a very ingeniously constructed die, with two or three blows of the hammer. Remnants of old fillings and foil can be converted very readily into these caps.

Dr. Fisk has made a test of them about three years and reports well of them thus far. They can without doubt be used to good advantage in many cases; for instance, large crown cavities of the molars, or indeed any other cavities to which they may be conformed, that are to be filled with oxy-chloride of zinc. In using the cap, one should be selected of the proper size, and fitted as perfectly as possible to the orifice of the cavity. The cavity, having been prepared, is now filled with the oxy-chloride, into which, while it is plastic, the flange of the cap is forced until it comes down to its proper position and adaptation upon the orifice of the cavity. The filling should then be kept free from moisture, till it is thoroughly set, when the gold surface may be finished as desired.

What the result may be, as to permanence, it is certain that this arrangement will be much more enduring than filling of oxy-chloride alone. This method will be valuable in the hands of skillful operators. We presume the caps may be obtained at the dental depots or, at least, of Dr. Fisk; or, what would be better, send to him and get a die—its operation is so simple that any one can use it. The cost of the appliance we do not know, but it can not be much.

